# Lissencephaly and Advanced-Stage Congenital Cytomegalovirus Infection in a Neonate

**DOI:** 10.7759/cureus.61576

**Published:** 2024-06-03

**Authors:** Ahmira Jade E Manalac, Erika Lytle, Liaqat Khan, Koshy George

**Affiliations:** 1 Pediatrics and Neonatology, Edward Via College of Osteopathic Medicine, Monroe, USA; 2 Pediatrics, Edward Via College of Osteopathic Medicine, Monroe, USA; 3 Neonatology, Rapides Regional Medical Center, Alexandria, USA

**Keywords:** premature neonate, prematurity complications, high risk neonates, classic lissencephaly, congenital cytomegalovirus infection

## Abstract

This case report investigates the management of a 24-week-old neonate with congenital cytomegalovirus (CMV) infection and its sequelae, including severe intrauterine growth restriction, thrombocytopenia, and brain anomalies, ultimately progressing to lissencephaly. The diagnostic challenges included delayed clinical suspicion of congenital CMV, which was not identified until after delivery through CMV DNA polymerase chain reaction, and differentiating its symptoms from other potential causes of the neonate’s condition. Aggressive interventions included antibiotics, antiviral therapy with ganciclovir, and supportive measures such as intubation, CPR, respiratory support, blood transfusions, and management of coagulopathy. Despite these efforts, the patient deteriorated due to progressive hypoperfusion, hypoxemic cardiorespiratory failure, and disseminated intravascular coagulopathy. Due to the poor prognosis and extent of multiorgan damage, support was withdrawn per parental consent. This case highlights the complications encountered when managing an advanced-stage neonatal CMV infection and emphasizes the importance of a multidisciplinary and holistic approach to guide diagnosis and treatment.

## Introduction

Congenital cytomegalovirus (CMV) poses a significant concern to universal public health matters. A member of the Herpesviridae family, CMV has the propensity to cause a sequela of congenital abnormalities when transmitted from the mother to the fetus during pregnancy [1]. Acute onset of CMV infection can present with vague signs, such as fever, sore throat, and fatigue, or may present asymptomatically. Thus, the pregnant mother may present as entirely asymptomatic, whereas congenital CMV has several repercussions on the fetus, including hearing loss, seizures, and cognitive developmental delay [2]. Although there is no definitive cure for CMV infection, there are medications available to slow the reproduction of the virus. When CMV infection is detected prenatally, interventions with valganciclovir (VGCV) and CMV hyperimmune globulin have produced encouraging outcomes by reducing viral load and transmission to the fetus [3]. However, it is important to note that many individuals may not even be aware they are infected with CMV unless they undergo comprehensive testing, which is atypical of routine prenatal testing. Among the general pregnant patient population, recommendations primarily focus on risk reduction strategies, such as avoiding contact with urine and saliva from infants and young children. Despite these precautions, the risk of infection cannot be entirely eliminated. As Manicklal et. al. described in their study, the “silent" nature of CMV infection during pregnancy emphasizes the need for further studies and testing, education, and awareness for expectant mothers, and the development of effective preventative measures [2].

Congenital CMV is estimated to affect 0.2-2.2% of live births worldwide [2]. In a 17-year study, Bristow et al. examined a staggering total of 777 infant deaths in the United States due to CMV infection manifestations, with 557 being under the age of one year old [4]. Furthermore, children surviving with severe long-term disabilities require specialists and ongoing medical care, which imposes a significant burden on healthcare costs while also profoundly affecting patient and caretaker quality of life. While CMV is a prominent cause of sensorineural deafness and intellectual disability globally, the CDC does not recommend routine screening given that most CMV testing is challenging to interpret, as it primarily detects initial infections and does not ascertain fetal infection status [5]. Testing for CMV IgG prenatally is only typically conducted if a pregnant woman exhibits symptoms resembling mononucleosis or if fetal anomalies, raising clinical suspicion, are detected on routine ultrasound. Furthermore, an amniocentesis must be performed to confirm fetal infection after 21 weeks of gestation [3]. Even so, barriers such as lack of awareness, perceived importance, or access to prenatal care may prevent some women from seeking the testing required for CMV diagnosis.

Current guidelines for managing congenital CMV include two to six weeks of IV ganciclovir, followed by a transition to oral VGCV for patients with severe, life-threatening symptoms. Patients without life-threatening presentations are treated with oral VGCV for six weeks. During this treatment period, it is essential to closely monitor the absolute neutrophil count, platelet count, blood urea nitrogen, creatinine, and liver function tests to ensure that multiorgan damage does not occur [6].

This case report aims to explore the challenges of managing a neonate with congenital CMV infection. Due to the nature of CMV’s multisystemic manifestations, our presentation underscores the importance of consulting medical teams across multiple disciplines to optimize evidence-based healthcare. By illustrating a real-life scenario, this case provides insight into management for affected infants and contributes to other existing literature regarding congenital CMV and its clinical manifestations.

## Case presentation

A 355-g African-American female was born via urgent cesarean section at 24 weeks gestation due to fetal heart rate decelerations. The mother’s blood type was B positive. The pregnancy was complicated by intrauterine growth restriction and absent end-diastolic flow. Antenatal steroids were administered for expectant preterm delivery. Upon delivery of the fetus, immediate intubation was required, and brief CPR was administered. Apgar scores were 4, 5, and 8 at one, five, and 10 minutes, respectively. Following the stabilization of vital signs, the patient was subsequently transferred to the NICU for further monitoring and management.

A sepsis screen was completed due to suspected meconium-stained fluid, including a complete blood count, a methicillin-resistant Staphylococcus aureus screen, a polymerase chain reaction (PCR) culture, a blood culture from a cord sample, and lactate levels. The patient was started on empiric antibiotics while the blood cultures were pending. Furthermore, a newborn screen and CMV DNA PCR were completed due to severe intrauterine growth restriction, thrombocytopenia, and a suspected brain anomaly. The results of the patient’s preliminary testing are displayed in Table 1.

**Table 1:** Patient's neonatal laboratory results at one day of life

Laboratory parameter	Result	Normal range
Leukocyte count (K/uL)	6.5	9.0-34.0
Hemoglobin (g/dL)	11.4	13.5-20.5
Hematocrit (%)	38	41-73
Platelet count (K/uL)	42	150-450

**Figure 1:**
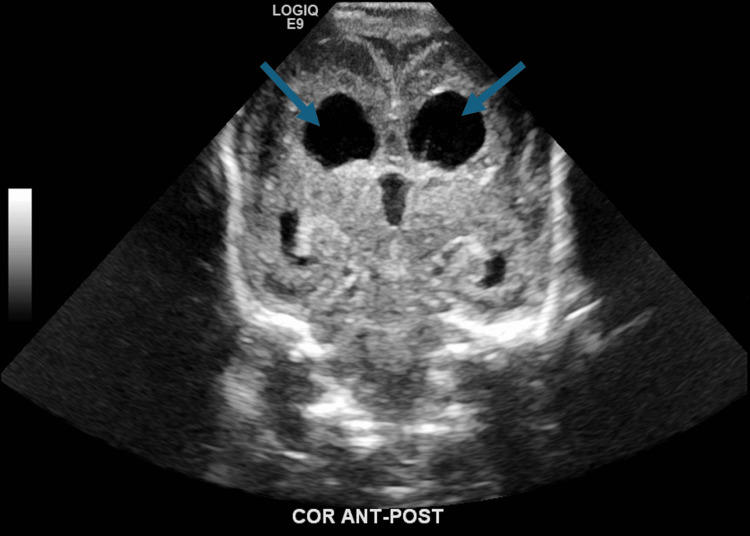
Still image from the head ultrasound on day of life seven with evident congenital abnormality of the midline structures noted with secondary prominence of the ventricles (blue arrows)

Neonatal laboratory results revealed leukopenia, anemia, thrombocytopenia, and positive CMV DNA PCR. At day of life seven, the head ultrasound revealed congenital abnormality of the midline structures noted with secondary prominence of the ventricles, as seen in Figure 1.

**Figure 2:**
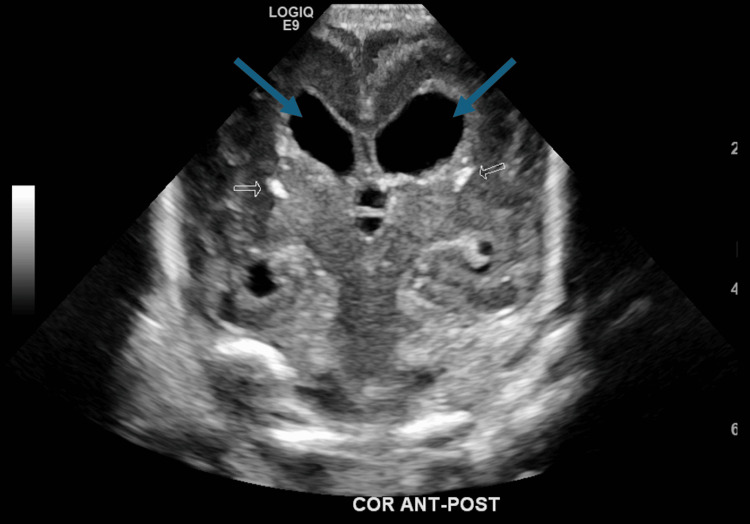
Still image from the head ultrasound on day of life 27 displaying stable ventriculomegaly (blue arrows) and hyperechoic periventricular foci observed bilaterally (white arrows) and prominently seen compared with the previous head ultrasound

**Figure 3:**
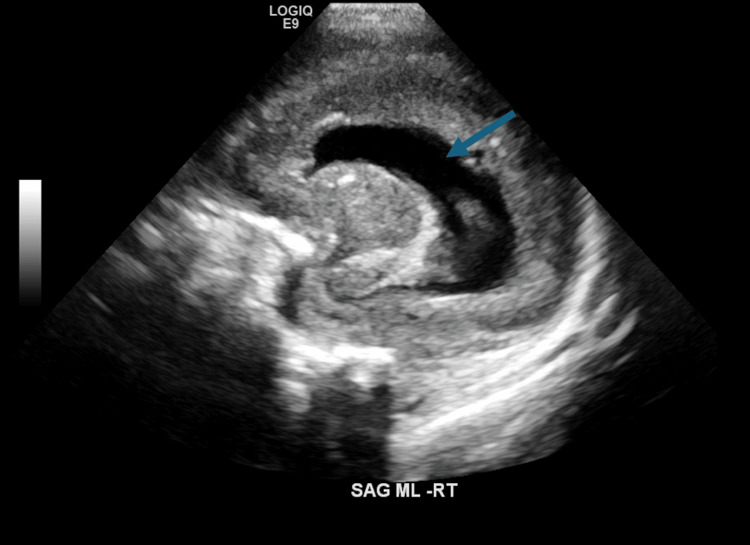
Still image from the head ultrasound on day of life 27, suggestive of an absent corpus callous (blue arrow)

At 27 days of life, the head ultrasound was repeated and revealed brain anomalies associated with congenital CMV infection. Brain abnormalities can be seen in congenital CMV infection with ventriculomegaly and periventricular calcification and the suggestion of critical dysplasia or migrational abnormalities, previously not noted in the prior ultrasound, as seen in Figure 2 and Figure 3.

**Figure 4:**
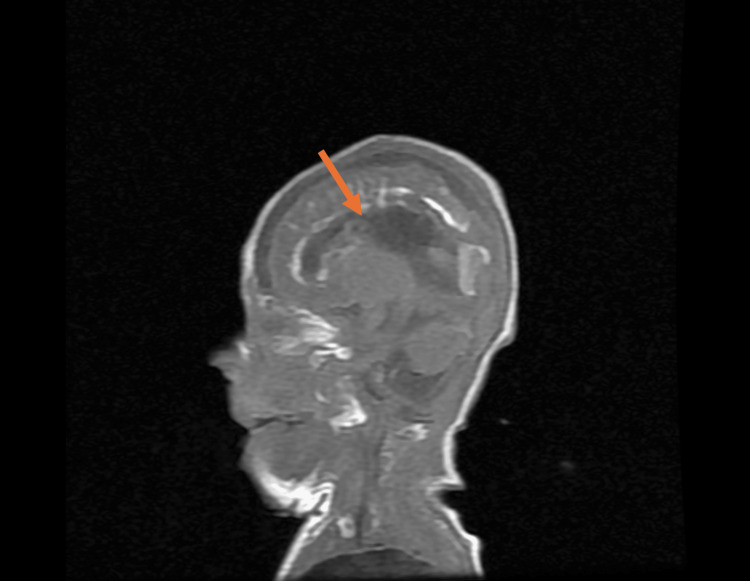
Sagittal plane of brain MRI at day of life 31, displaying agenesis of corpus callosum (orange arrow)

**Figure 5:**
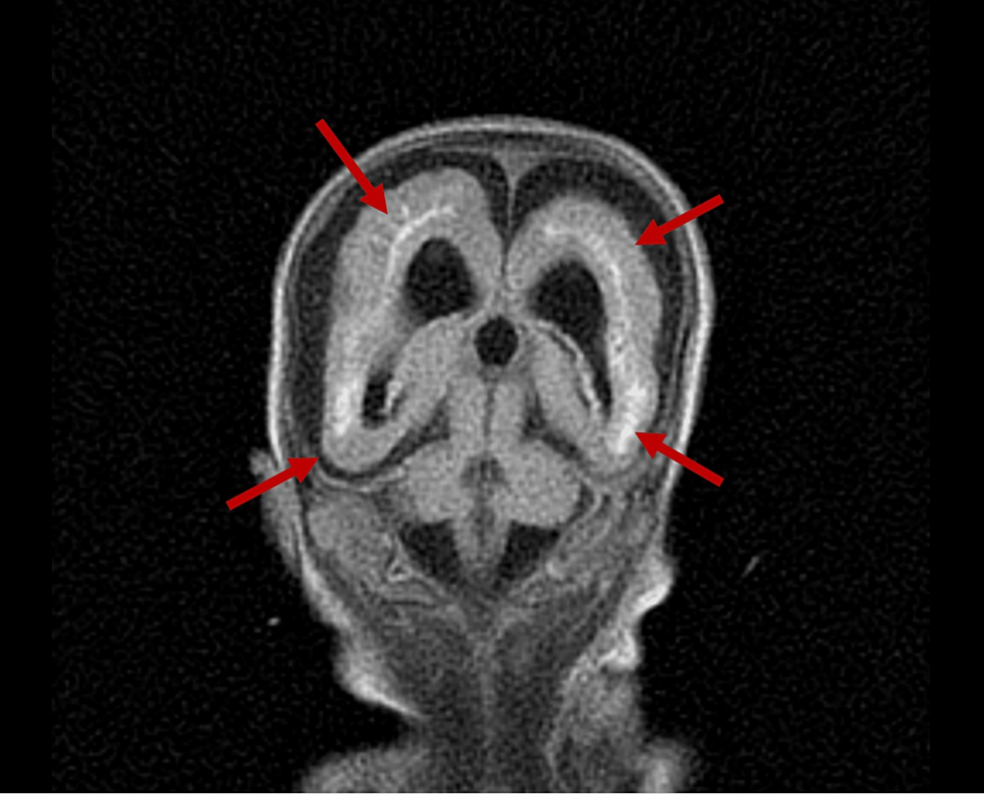
Coronal plane of brain MRI at day of life 31, displaying generalized brain calcifications (red arrows)

**Figure 6:**
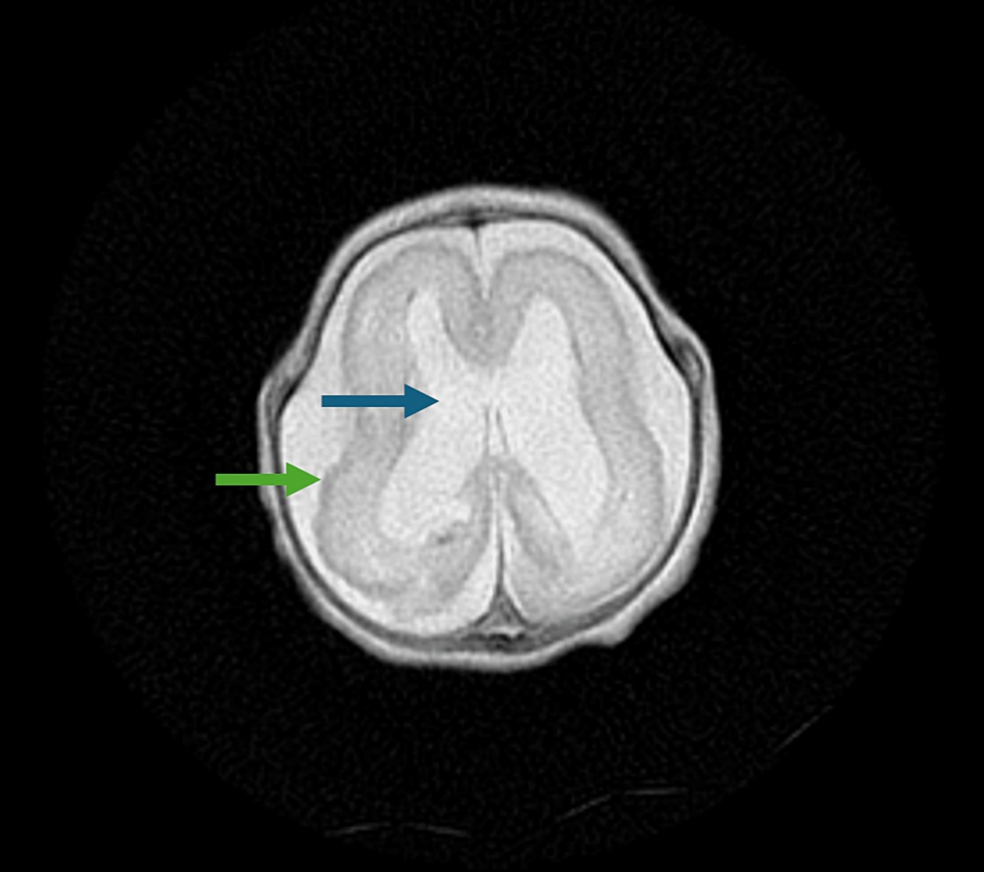
Axial plane of brain MRI at the day of life 31, displaying progression to lissencephaly (green arrow) and Grade 2 intraventricular hemorrhage with occipital horn-dependent blood (blue arrow)

**Figure 7:**
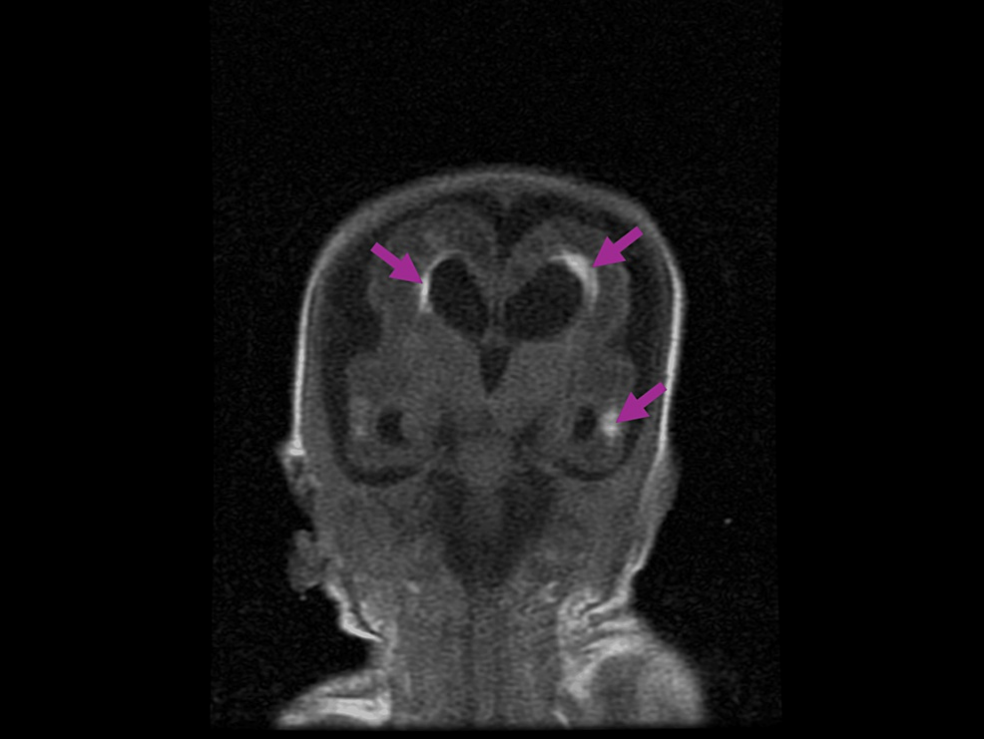
Coronal plane of brain MRI at day of life 31, displaying scattered gloss and scarring of the brain (purple arrows)

The MRI of the brain was completed on day of life 31. The brain MRI revealed global delayed maturation of the brain with scattered gliosis and scarring, Grade 2 intraventricular hemorrhage with occipital horn-dependent blood vessels, and generalized brain calcifications with evidence of structural damage, appearing to progress to lissencephaly. Still images of the patient’s brain MRI are seen in Figure 4, Figure 5, Figure 6, and Figure 7.

Treatment included caffeine for apnea, high-flow oscillatory support, and multiple blood transfusions. Antibiotic therapy was initiated, whereby IV vancomycin and IV piperacillin-tazobactam were administered alongside IV ganciclovir at 6 mg/kg/dose every 12 hours for 16 days to treat the CMV infection. Seizure-like activity was noted once the patient was 15 days old; thus, due to the history of lissencephaly, phenobarbital was administered at 4 mg/kg every 12 hours.

At 32 days of life, the patient began to have bloody stools and was placed on a nothing-by-mouth status. A septic workup was initiated, and IV vancomycin/piperacillin-tazobactam was administered. A platelet transfusion was administered to treat evidence of thrombocytopenia. Subsequently, the patient developed progressive hypotension, thus requiring dopamine therapy. Over the next few hours, the patient developed disseminated intravascular coagulation, prompting infusions of packed red blood cells and fresh frozen plasma. Despite fluid resuscitation and epinephrine treatment, the patient’s status declined with progressive hypoperfusion and a hypoxemic cardiorespiratory failure response. Kidney, ureter, and bladder ultrasound revealed questionable pneumatosis. Given the profound brain damage and deteriorating condition, parental consent was obtained to withdraw life support at 32 days of life.

## Discussion

Management of congenital CMV in neonates poses significant challenges, especially in the setting of extreme prematurity. Thus, early detection and intervention must take place to optimize outcomes in the affected neonates [7]. In the presented case, although the mother received prenatal care, clinical suspicions of congenital CMV did not arise until after delivery. Congenital CMV was subsequently detected via CMV DNA PCR testing per current guidelines [7-9]. Current management guidelines surrounding congenital CMV infection were closely considered and followed in this case, whereby prompt administration of antiviral therapy with IV ganciclovir was administered [7,9-11]. Moreover, studies currently show that ganciclovir therapy improves outcomes for sensorineural hearing loss and neurodevelopmental complications in CMV infection, thus highlighting the importance of promptly initiating comprehensive screening and diagnosing neonates at risk [12].

A peculiar finding with our patient was that she developed lissencephaly, a condition characterized by a smooth brain surface resulting from disruptions in fetal brain development. CMV infection is implicated in lissencephaly, as CMV pathogenesis interferes with the migration of neurons during this crucial developmental stage, directly affecting neural progenitor cells and impeding their normal function [13]. Additionally, CMV infection induces an inflammatory response in the fetal brain. This inflammatory cascade can result in secondary effects like hypoxia or ischemia in the developing brain, further exacerbating the condition [13]. Collectively, these factors contribute to the detrimental effects associated with lissencephaly. Current treatment guidelines recommend supportive and symptomatic measures, including anticonvulsant therapy, to prevent or control active seizures [14]. However, once the patient has progressed to this stage, there is no cure available.

Recognizing the urgency to detect CMV infection earlier and implement aggressive treatments to prevent progression to lissencephaly, researchers and developers must focus on these crucial objectives. Although diagnostic and treatment modalities are available for treating congenital CMV, there are still challenges that remain. For example, there is still a need to have readily available, universally effective vaccines and a more comprehensive range of antivirals suitable for neonates. One notable study by Bernstein et al. demonstrated the ability to induce robust immune responses against CMV, suggesting its potential to prevent congenital CMV infection in pregnant women and reduce the risk of transmission to their babies [15]. Thus, future testing methods for acute CMV infection must be readily accessible for pregnant women and must ensure timely detection, regardless of whether the patient has had a previous infection with CMV. Moreover, the method must be highly effective in providing prompt results to prevent the development of long-term sequelae associated with congenital CMV infection.

To improve outcomes for affected neonates, ongoing research is vital to expand screening programs for congenital CMV infections [1,2,7,9,16-18]. In Turner et al.’s study involving infants with very low birth weight, researchers screened each participant for congenital CMV. Similar to our case, they found that the primary outcome for these cases was death. Other morbidities included neurological injury, sensorineural hearing loss, and developmental delay. The study recommended routine CMV screening for mothers and infants with very low birth weight at risk and suggested conducting large-scale studies on the efficacy and safety of current congenital CMV treatment guidelines [19]. Furthermore, as Chiereghin et al. concluded in their research, without implementing universal screening for neonatal CMV, certain infants who contract the infection and subsequently develop delayed neurological complications may lose the opportunity to receive timely instrumental and therapeutic interventions aimed at mitigating or addressing CMV-related illnesses [20].

Management of congenital CMV in neonates must involve a multidisciplinary team including, but not limited to, neonatology, infectious disease, and other specialists. This interdisciplinary team can collaborate to create the best, holistic support for both the neonate and the family, ensuring that all aspects of the neonate’s care, including developmental and psychosocial, are addressed. The case at hand required a multidisciplinary team due to the complex nature of the patient’s condition and the variety of medical interventions needed. The patient’s need for respiratory support, blood transfusions, and management of coagulopathy involved coordination between neonatal intensivists, respiratory therapists, hematologists, and transfusion medicine specialists. Furthermore, radiology’s assessment of the imaging studies, particularly the detection of brain anomalies, played a crucial role in raising clinical suspicion of congenital CMV infection and guiding the future direction of the patient’s treatment.

Ethical applications, such as informed parental consent, are crucial when considering treatment plans and medical decision-making [7,17,18]. In this case, the decision to withdraw life support was made after an extensive discussion between the physician and the parents. These decisions were made in the neonate’s best interest while honoring parental rights. By acknowledging the delicate balance between aggressive medical treatments and the potential impact on the neonate's quality of life, healthcare providers can navigate ethically challenging situations with compassion and empathy, ensuring that the care provided aligns with the values and preferences of both the neonate and their family.

## Conclusions

The management of congenital CMV infection in neonates demands a multifaceted approach involving early detection, aggressive treatment, and comprehensive support systems. Despite the challenges posed by extreme prematurity and the complexity of associated conditions like lissencephaly, advancements in diagnostic techniques, therapeutic modalities, and vaccination strategies offer hope for improved outcomes. However, ongoing research, expanded screening programs, and interdisciplinary collaboration are essential to address remaining gaps and challenges. By prioritizing these efforts and upholding ethical principles, healthcare providers, researchers, and policymakers can work together to mitigate the impact of congenital CMV infection and enhance the quality of care for affected neonates and their families.
